# Nickel Cobalt Sulfide core/shell structure on 3D Graphene for supercapacitor application

**DOI:** 10.1038/s41598-017-02309-8

**Published:** 2017-05-18

**Authors:** Lemu Girma Beka, Xin Li, Weihua Liu

**Affiliations:** School of Electronics and Information Engineering Xi’an Jiaotong University, Shaanxi, 710049 China

## Abstract

Three-dimensional (3D) core/shell structure of nickel cobalt sulfide is nano-engineered by using series of hydrothermal steps on a CVD grown graphene for supercapacitor application. This core/shell is composited of NiCo_2_S_4_ nanotube (NCS) as core and Co_x_Ni_(3−x)_S_2_ (CNS) nanosheets as a shell. The as-synthesized composite exhibits excellent electrochemical properties by using the advantage of NCS nanontube core as superhighway for electron and ion transport, and CNS nanosheets shell as high active area pseudocapacitive material. The 3D graphene layer serves as excellent surface area to support 3D NCS/CNS; moreover, it provides excellent electrical conductivity between nickel foam current collector and the 3D NCS/NCS composite. Using these hybrid advantages the as-synthesized graphene/NCS/CNS composite electrode exhibits high areal capacitance of 15.6 F/cm^2^ at current density of 10 mA/cm^2^; excellent cycling stability of 93% after 5000 of cycles and excellent rate capability of 74.36% as current increase from 10 to 100 mA/cm^2^. Moreover, a prototype of asymmetric device fabricated using graphene/NCS/CNS as positive electrode and RGO as negative electrode exhibits high energy density of 23.9 Wh/kg and power density of 2460.6 W/kg at high operating current of 100 mA. Such high performance electrode material may get great application in future energy storage device.

## Introduction

The advancement in use of portable electronic devices and high standards of living imposed the world to consume large amount of energy. So, to overcome this demand designing highly efficient energy storage device is an important issue. Among the energy storage devices, supercapacitors (SCs) are one of the promising devices due to their ultra-high power density, long cycling stability, wide range of operating temperature, and improved safety^1–5^. Based on energy storage mechanism supercapacitors are of two types, namely, electrical double-layer capacitors (EDLCs) in which energy storage is dominated by electrostatic charge diffusion and accumulation at the interface of the electrode and electrolyte; and pseudocapacitors in which energy storage is by reversible Faradaic reactions that occur at the electrode surface^6^.

It is well known as the performance of SCs devices mainly depends on the properties of the materials used; and the key to achieve outstanding performance is to seek appropriate materials and designing suitable electrode architecture that support maximum efficiency of the proposed material. Recently, nanostructured nickel cobalt sulfides’ composites have attracted much research attention as pseudocapacitor electrode materials^7, 8^. Different morphologies of ternary metal sulfides were investigated as high performance psudocapcitive materials (nanotube, urchin, nanosheet, cubic and flower)^9–13^. Even though ternary metal sulfides showed promising performance, practical application of ternary metal sulfides as electrode material is still limited because of two main challenges, namely, low rate performance at high current densities and short cycling stability.

In general to address these kinds of problems a wide range of nanomaterial design concepts have been developed such as decreasing the material features size to nanoscale with the structures such as core/shell, hallow particles and tubes^14, 15^. Recently, considerable effort has been devoted in synthesizing core/shell nanostructures for energy storage applications to increase the active area, cycling stability and rate capability of the device. For example, Kong *et al*.^16^ reported Core-shell NiCo_2_S_4_ nanostructures supported on nickel foam with capacitance value of 1.948 F/cm^2^ at 1 mA/cm^2^ and 79.36% of rate retention at 20 mA/cm^2^; Fu *et al*.^17^ reported Cobalt sulfide nanosheets coated on NiCo_2_S_4_ nanotube arrays as core/shell structure with highest capacitance of 4.74 F/cm^2^ at 5 mA/cm^2^ and rate capability of 47.67% at current density of 50 mA/cm^2^; similarly, Zhang *et al*.^18^ has reported nanoforest of hierarchical Co_3_O_4_@NiCo_2_O_4_ nanowire arrays synthesized via a facile strategy for electrochemical supercapacitors. Unfortunately, the reported structures still suffer from low capacitance, inferior rate performance and low utilization rate of active materials and low cycling performance at high currents. Even though electrode materials possessing high capacity, long cycling stability, and excellent rate capability are highly desirable, it is difficult to achieve all these objectives simultaneously from a single materials. So, hybridizing of different materials with different properties is becoming interesting research area^5, 19^. Recently, hybridizing ternary metal sulfides with nanocarbon based material is becoming important research area. From nanocarbons, graphene is one of hot research area for energy storage application due to its unique properties, including high surface area, excellent conductivity and excellent mechanical and chemical stability which are desirable properties for SCs application^20, 21^. In ternary metal sulfide/graphene hybrid composite, graphene serve as flexible high surface area to physically support pseudocapacitive ternary metal sulfides and excellent electron transport channels and resulted in improved capacitance, cycling stability and rate capability. Du *et al*.^22^ demonstrated facile synthesis and superior electrochemical performances of CoNi_2_S_4_/graphene nanocomposite suitable for supercapacitor electrodes with maximum specific capacitance of 2009.1 F/g at a discharge current density of 1 A/g; Wang *et al*.^23^ reported electrodeposited nickel cobalt sulfide nanosheet arrays on 3D graphene/nickel foam for high performance supercapacitors with good electrochemical performance; similarly, Patil *et al*.^24^ and Beka *et al*.^25^ reported the improvement in capacitance performance after hybridizing of ternary metal sulfides with graphene. Even though there are many works mentioned in the literature for ternary metal sulfide/graphene composite, the electrochemical performance is still in its infancy compared to the theoretical expectations. As a result, design of an integrated smart architecture ternary metal sulfide porous nanostructure that consists of 3D building blocks grown on 3D graphene is significantly desired, to use synergistic advantages of graphene and the ternary metal sulfides.

Herein, we proposed a porous 3D nickel cobalt sulfide core/shell (NCS/CNS) nanostructure grown on 3D graphene decorated nickel foam for supercapacitor applications. Initially, the 3D graphene was grown on nickel foam using CVD process; then, using series of hydrothermal treatments 3D NCS/CNS core/shell is grown on the graphene layer. This hybrid composite uses the high surface area and excellent mechanical properties of graphene to support 3D porous NCS/CNS core/shell; moreover, the excellent conductivity of graphene leads to super electron transport channels between the collector and the active material. The 3D NCS/CNS core/shell nanocomposite contains numerous nanosheet shells which results in excellent porosity and high active area which are favorable for energy storage application. Using these synergistic effects of graphene and ternary metal sulfides the as-fabricated nanocomposite structure shows excellent specific capacitances with outstanding capacitance retention at high current densities and excellent cycling stability. To further evaluate our electrode material for practical application we assembled a prototype asymmetric device of 3D NCS/CNS core/shell/on graphene decorated nickel foam as positive electrode and RGO as negative electrode, and the assembled device receives an excellent electrochemical performance. The results obtained in this nanocomposite showed promising performance of hybrid structure of porous core/shell ternary metal sulfide and graphene for future energy storage applications.

## Results and Discussions

### Structural characterization

**Figure 1:**
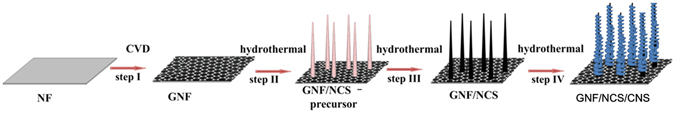
Schematic representation of growth process.

**Figure 2:**
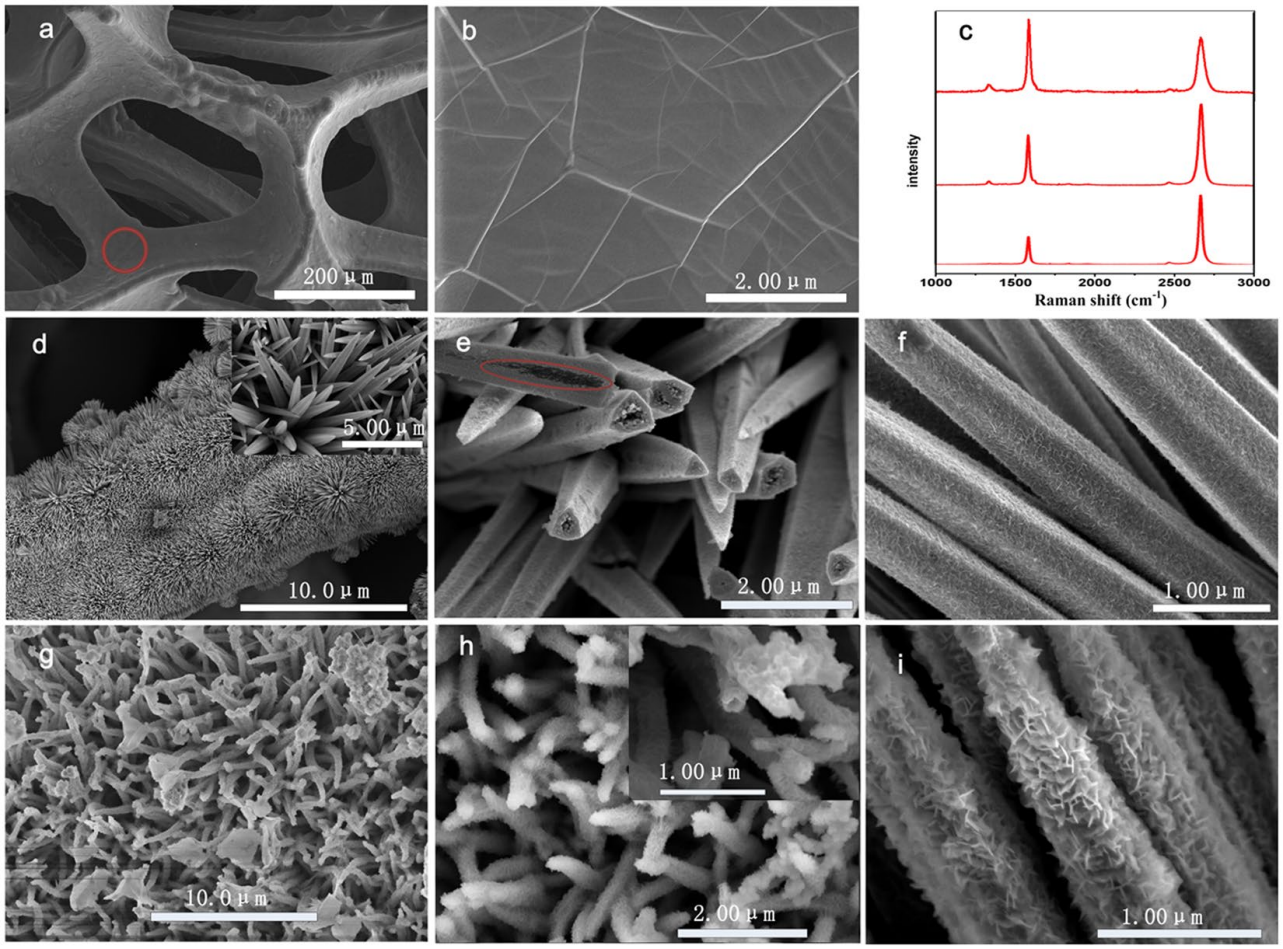
(**a**) SEM image of graphene on 3D nickel foam, (**b**) high magnification SEM image of graphene on circle marked area of (**a**), (**c**) Raman spectroscopic test results of graphene on nickel foam at different places, (**d**) GNF/NCS-precursor on 3D nickel foam, (**e**) and (**f**) GNF/NCS after the second hydrothermal treatment, (**g**–**i**) GNF/NCS/CNS core/shell structure at different magnifications.

The fabrication process of our composite electrode is schematically represented in Fig. 1. Step I, a 3D graphene was grown on nickel foam using CVD process and labeled as GNF and used as substrate for the growth of pseudocapacitive material. Step II, NCS nanoneedle precursor was uniformly grown on the GNF using hydrothermal process, and the result is labeled as GNF/NCS precursor. Step III, NCS precursor nanoneedles are transformed to NCS nanotube using Kirkendall effect^26^ and labeled as GNF/NCS nanotube. Lastly, CNS porous nanosheets were grown on the NCS nanotube and the sample is labeled as GNF/NCS/CNS. Figure 2 illustrates a typical SEM images and Raman test results of as prepared sample. Figure 2a shows the SEM image of CVD grown graphene on 3D nickel foam. Figure 2b shows the magnified SEM image of graphene at circle marked area of Fig. 2a. The graphene grown on nickel by using CVD method is slightly disordered and non uniform thickness as characterized by Raman spectroscopy^27^. However, the electronic and mechanical properties of graphene are strongly influenced by the thickness or number of layers of graphene present in the composite. As a result, the dimensional characterization of the as-synthesized graphene on nickel foam is crucial. So, to know the average thickness of our graphene we conducted Raman spectroscopy test on different places and almost the sample shows approximately any of the three graphs shown in Fig. 2c. As we can see form Fig. 2c there are small variations in intensity of 2D and G bands, so based on these band intensities we can say the graphene on our nickel foam is few layer graphene (less than 5 layers)^28^.

Figure 2d shows the SEM image of NCS nanneedles precursor uniformly and densely grown on GNF. The typical SEM image of GNF/NCS nanotube (Fig. 1 step III) is shown in Figure 2e. Clearly, it illustrates that the as-synthesized NCS nanoneedle precursor sample shown in inset Fig. 2d is converted into a nanotube structure after the second hydrothermal treatment using kirkendall an ion exchange reaction. Moreover, as it can be seen from the red marked place of Fig. 2e each nanoneedle has internally well defined nanotube structure; however, the nanotube is observed only if the tip nanoneedl is broken. The high-magnification SEM image of the outer surface of NCS nanotube is shown in Fig. 2f. Figure 2g–i show NCS nanotubes uniformly covered by ultrathin nanosheets forests illustrating the transformation of NCS nanotube into ultra rough GNF/NCS/CNS core/shell structure (step IV, Fig. 1). Clearly, the core/shell structure exhibits a dense and uniform porous nanosheet forest morphology grown on the surface of the nanontubes when compared to the NCS nanotube morphology of Fig. 2e and f. As it can be seen from Fig. 2i, the morphology of the composite becomes 3D after the core/shell formation which is very interesting for charge storage because of the introduction of more active area and excellent porosity on the surface which resulted easy diffusion of electrolyte ions. The growth mechanism of this nanosheet shell structure is illustrated as follows. Initially, the rough surfaces of NCS nanontubes (shown in Fig. 2f) are used as a template for nucleation of CNS nanosheets as a shell during the third step hydrothermal process. Therefore, once the nucleation center was generated on these porous areas, it commences the growth of CNS nanosheets at the vertical and planar direction and forms shell surface.

**Figure 3:**
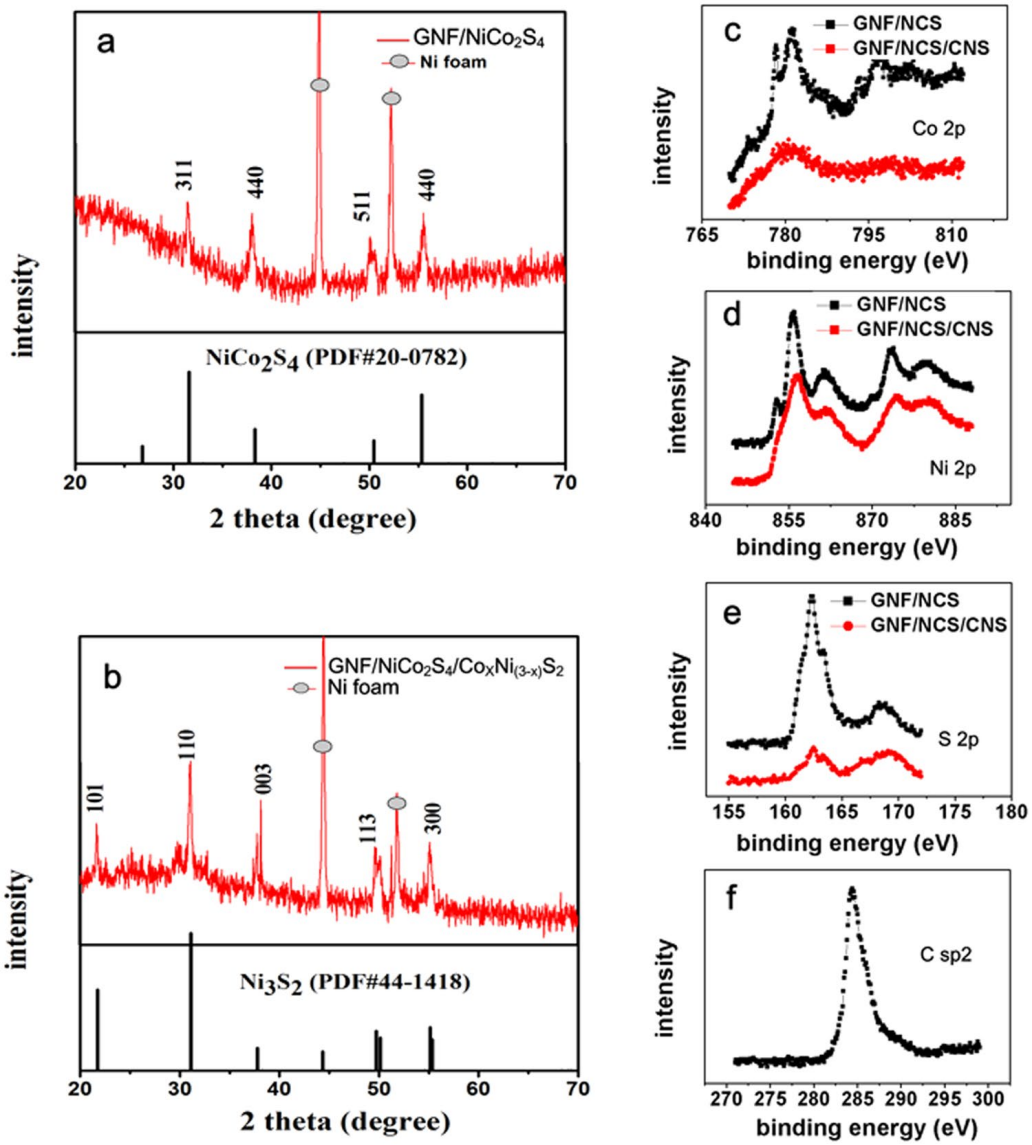
XRD diffraction patterns and XPS analysis (**a**) XRD diffraction pattern of GNF/NiCo_2_S_4_ nanotube and (**b**) XRD diffraction pattern of GNF/NiCo_2_S_4_/Co_x_Ni_(3−x)_S_2_ core/shell structure and (**c**–**d**) XPS analysis of Co, Ni and S black color before formation of core/shell and red color after formation of core/shell and (**e**) XPS analysis of C.

XRD diffraction pattern was used to investigate the crystal phase of as synthesized samples. In Fig. 3a and b, the two strong diffraction peaks at 44.7° and 52.1° are indexed to (111) and (200) diffraction planes of nickel foam current collector, respectively. The other peaks in Fig. 3a shows the XRD pattern of GNF/NCS. Clearly, it shows distinct peaks at 31.6°, 38.3°, 50.5° and 55.3° which corresponds to (311), (400), (511), and (440) diffraction planes, which can be indexed to the cubic phase of NiCo_2_S_4_ (JCPDS 43–1477). On the other hand, the crystal phase of the sample after the formation of shell is illustrated by using Fig. 3b; the distinct peaks at 21.696°, 31.4°, 38.26°, 50.3°, and 55.4° corresponds to the (101), (110), (003), (113), and (300) diffraction planes, respectively, which can be indexed to Ni_3_S_2_ (PDF # 24–0334). From the XRD pattern of the as-synthesized composite Co atoms cannot be directly seen, this is because the atomic radii of Co and Ni are quite similar and the partial substitution of Ni atoms by Co atoms does not change the crystal structure of Ni_3_S_2_ except change in lattice parameters^29^. So, substituting some of the Ni atoms by the Co atoms the composite can be indexed to a chemical composite of a form Co_x_Ni_3−x_S_2_ where 0 < × < 3. Especially, the existence of additional peaks in the GNF/NCS/CNS core/shell composite compared to GNF/CNS sample proves the successful transformation of the nanotube structure into GNF/NCS/CNS core/shell structures.

**Figure 4:**
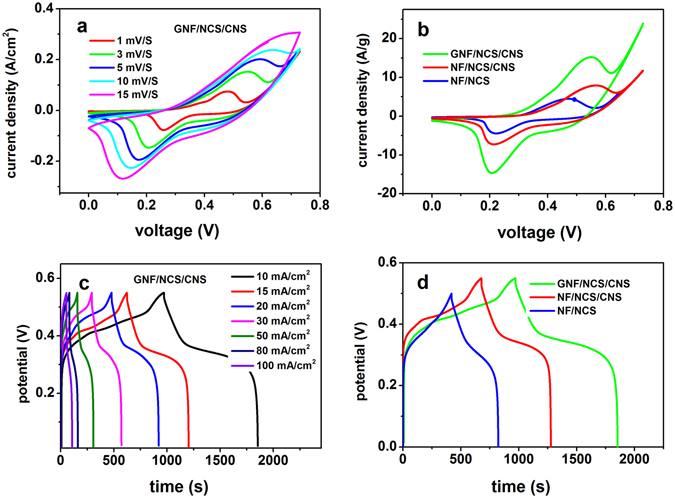
CV and GCD plots using three electrodes test results (**a**) CV plots of GNF/NCS/CNS core/shell at different scan rates, (**b**) comparison of CV plots of GNF/NCS/CNS core/shell, NF/NCS/CNS core/shell and NF/NCS at scan rate of 3 mV/S, (**c**) GCD plots of GNF/NCS/CNS core/shell at different current densities and (**d**) comparative GCD plots of GNF/NCS/CNS core/shell, NF/NCS/CNS core/shell and NF/NCS at current density of 10 mA/cm^2^.

The chemical states and electronic configuration of each element in the composite is studied in Fig. 3. Figure 3c shows the Co 2p XPS spectrum. The strong peaks at 781.4 and 796.5 eV corresponds to Co 2p_3/2_ and Co 2p_1/2_, respectively. The existence of 15.1 eV energy band difference between Co 2p_3/2_ and Co 2p_1/2_ spectrum confirms the existence of both Co^2+^ and Co^3+^ on the surface of the composite^30^. While in the core/shell composite the amount of Co is reduced and only one weak peak is observed at 781.4 eV for Co 2p_3/2_, suggesting the existence of only Co^3+^; the reduction in the cobalt amount is in agreement with the XRD analysis. Similarly, Fig. 3d depicts the Ni 2p XPS spectrum. The peaks at 855.6 and 873.1 eV corresponds to Ni 2p_3/2_ and Ni 2p_1/2_, respectively. The energy band difference between Ni 2p_3/2_ and Ni 2p_1/2_ spectrum is 17.5 eV which indicates the existence of both Ni^2+^ and Ni^3+^ on the surface of the composite before and after the formation of core/shell^31^; while the other peaks are satellite peaks. The S 2p XPS spectrum is shown in Fig. 3e. The binding energies at 163.5 and 162.4 eV correspond to S 2p_1/2_ and S 2p_3/2_, respectively^32^. In the core/shell composite the peaks are reduced due to less number of S atoms in the shell (Co_x_Ni_3−x_S_2_) compared to the number of S atoms in the core (Ni Co_2_S_4_). Finally, Fig. 4f reveals the C 2p spectrum; clearly, it shows one distinct peak at binding energy of 284.7 eV illustrating the existence of Sp^2^ hybridized carbon atoms forming honeycomb, implying existence of graphene in the composite^33, 34^.

### Electrochemical characterization

To measure the electrochemical performance of GNF/NCS/CNS core/shell structure we conducted comparative electrochemical tests with respect to control samples such as nickel foam (NF)/NCS/CNS core/shell and NF/NCS nanotube (without shell) structures which are grown on the pristine nickel foam (nickel foam without graphene). According to our previous report^35^ the capacitance of pristine graphene on nickel foam is very low and we didn’t consider in this paper for comparison. The CV and GCD plots of GNF/NCS/CNS core/shell and the respective comparison with control samples are shown in Fig. 4. Figure 4a shows the CV curves of GNF/NCS/CNS core/shell sample at various scan rates ranging from 1 to 15 mV/S. Obviously, a pair of well defined redox peak is observed within a potential window of 0 to 0.73 V demonstrating excellent pseudocapacitive characteristics of the as-synthesized sample. Figure 4b shows comparison CV test plots of GNF/NCS/CNS core/shell, NF/NCS/CNS core/shell and NF/NCS nanotube structures within a potential window range of 0 to 0.73 V at scan rate of 3 mV/S. As it can be seen clearly, the CV curves of GNF/NCS/CNS core/shell exhibits much higher area while NF/NCS nanaotube exhibits the least CV area. The GCD plots of as-synthesized GNF/NCS/CNS core/shell within potential window of 0 to 0.55 V at various current densities ranging from 10 to 100 mA/cm^2^ is shown in Fig. 4c. It can be seen that all the GCD curves are symmetrical at various current densities demonstrating excellent electrochemical reversibility and charge-discharge properties of as-synthesized electrode. Additionally, the GCD curves of our electrode shows a plateaus shape which is a typical pseudocapacitive behavior and well consistent with the CV peaks. Figure 4d, illustrates the comparative GCD plots of GNF/NCS/CNS core/shell, NF/NCS/CNS core/shell and NF/NCS structure. Obviously, GNF/NCS/CNS core/shell composite shows better area showing its superior performance which is in a good agreement with CV plots. As it can be seen from Fig. 4d the potential window of NF/NCS structure is 0.5 V while the core/shell composites can support a better potential window of 0.55 V, this is related to the increased active areas in case of core/shell due to the existence of plenty porous nanosheets which can store more charges.

**Figure 5:**
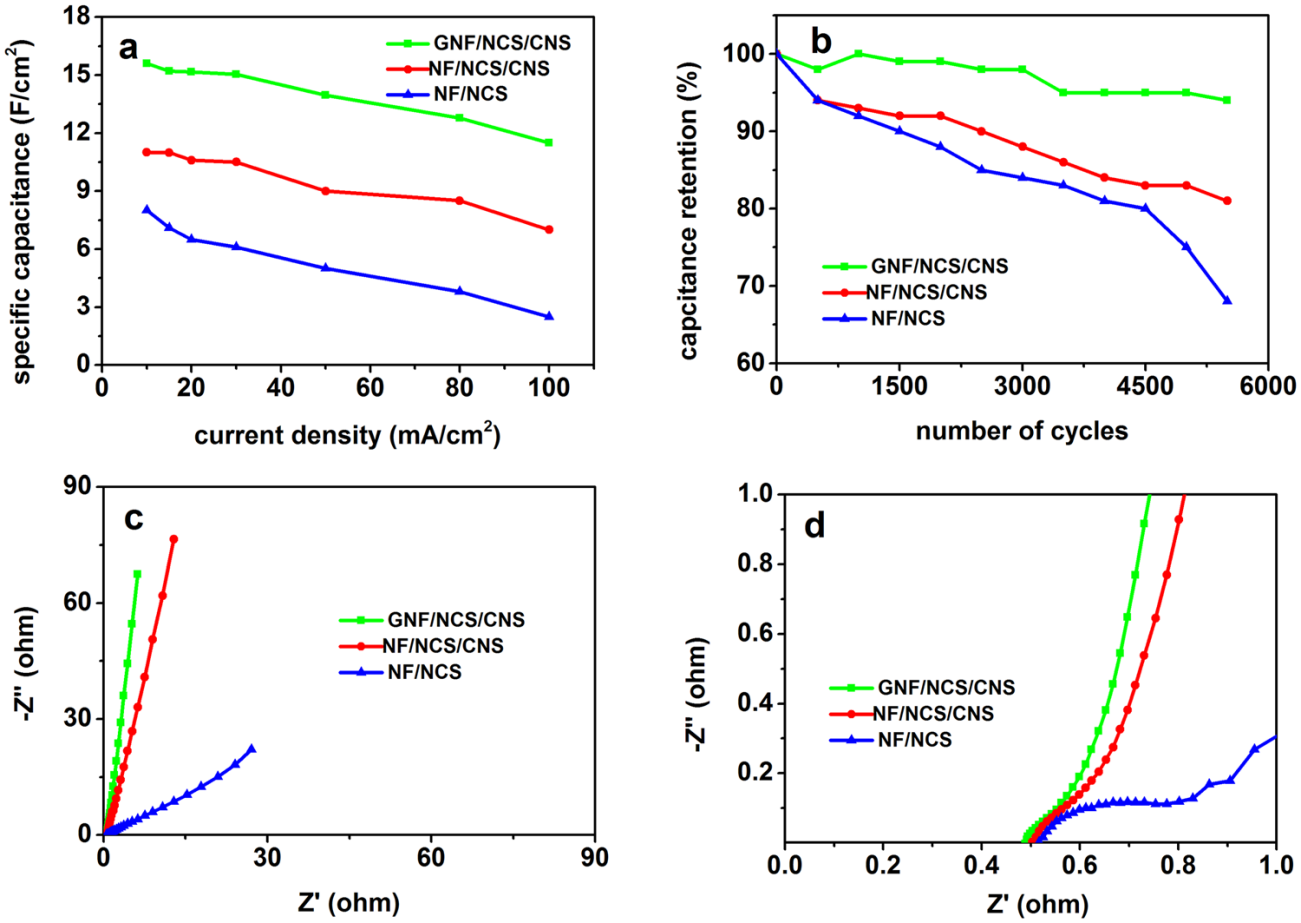
Electrochemical performance comparisons of GNF/NCS/CNS, NF/NCS/CNS and NF/NCS using three electrodes test (**a**) plots of areal capacitances at different current densities, (**b**) plots of capacitance retentions rate as number of cycles, (**c**) EIS plots (100 KHz–0.01 Hz) of composites, and (**d**) high-frequency region EIS plots.

The specific capacitances of the as-synthesized composites are calculated using equation (1) and (2). The highest capacitance obtained for GNF/NCS/CNS core/shell structure is 15.6 F/cm^2^ at current density of 10 mA/cm^2^. For more detail, Table 1 shows list of calculated specific mass capacitance (F/g) and specific areal capacitance (F/cm^2^) of GNF/NCS/CNS core/shell at different current densities. Even at high current density of 100 mA/cm^2^ our porous GNF/CNS core/shell shows high areal capacitance of 11.6 F/cm^2^. Thus, the as synthesized structure shows 74.36% capacitance retention as current increase from 10 to 100 mA/cm^2^. On the other hand, the NF/NCS/CNS core/shell and NF/CNS core show the highest specific capacitance of 10.9 and 8 F/cm^2^ at current density of 10 mA/cm^2^, respectively. Moreover, Fig. 5a shows the capacitive performance comparison of GNF/NCS/CNS core/shell, NF/NCS/CNS core/shell and NF/NCS samples at different current densities. Clearly, the composites with core/shell structure show excellent capacitance performance with excellent rate performance. This is due to the contribution of high surface area graphene which supported 3D porous NCS/CNS core/shell structure with plenty of nanosheets on the surface of the core structure. These porous shells structures serve as electrolyte reservoirs; these internal electrolyte reservoirs will bring the internal active surface layers directly into contact with the electrolyte and significantly decrease the distance of electrolyte ion diffusion and it leads to easy diffusion of electrolytes into active areas even at high current densities and leads to high rate capability. Such high areal capacitance at high current density proves great performance of our core/shell structure. Moreover, the graphene layer sandwiched between the NCS/CNS core/shell and nickel foam current collector serves as a super high way for electron transport and leads to better rate performance. Interestingly, using these synergistic advantages of graphene and NCS core/shell 3D porous structure our GNF/NCS/CNS core/shell evidently shows superior performance compared to most of recent reported core/shell structures as shown in Table 2.

**Table 1:** Specific and areal capacitances of GNF/NCS/CNS core/shell at different currents.

I(mA)	10	15	20	50	80	100
C_A_(F/cm^2^)	15.6	15.2	15.05	14	12.77	11.6
C_s_(F/g)	1950	1900	1881	1750	1596	1437.5

**Table 2:** Comparison of electrochemical performance of our GNF/NCS/CNS core/shell structure with recently reported literatures.

Type of electrode	Capacitance	Energy density	Power density	Cycles	Ref.
NiCo_2_S_4_@MnO_2_	2.6 F/cm^2^, 3 mA/cm^2^	—	—	52,3000, 50 mV/S	39
NiCo_2_S_4_@MnO_2_	1337.8 F/g, 2.0 A/g	—	—	82, 2000	39
Co_3_O_4_@Co_3_S_4_	1284.3 F/g, 2 mV/S	1.5 mWh/cm^3^	6.1 W/cm^3^	93.1, 5000	40
NiCo_2_O_4_@Ni_3_S_2_	1716 F/g, 1 A/g	—	—	83.7%,2000, 4	41
NiCo_2_S_4_/NiCo_2_S_4_	1.948 F/cm^2^, 1 mA/cm^2^	22.8 wh/kg	160 w/kg	94,5000, 2	16
Cobalt sulfide nanosheets coated on NiCo_2_S_4_ nanotube arrays	4.74 F/cm^2^, 5 mA/cm^2^	—	—	76.1, 1500, 50	17
Graphene/NiCo_2_S_4_/Co_x_Ni_(3−x)_S_2_	**15.6 F/cm** ^**2**^ **, 10 mA/cm** ^**2**^ **or 1950 F/g, 1.25 A/g**	**29.9 wh/kg**	**2460.6 W/kg**	**93,5000**	**This work**

Cycling stability is one of key parameter to evaluate the performance of SCs. As a result, we conducted a comparative cycling stability test for GNF/NCS/CNS core/shell, NF/NCS/CNS core/shell and NF/NCS core electrodes by applying constant current density of 50 mA/cm^2^ and the result is shown in Fig. 5b. Clearly, the GNF/NCS/CNS core/shell sample showed better stability of 93% after 5000 cycles of charge discharge while NF/NCS/CNS core/shell and NF/NCS core showed 82 and 71%, respectively under similar cycle numbers. Obviously, the core/shell structure show better performance compared to the pristine core sample while the GNF/NCS/CNS core/shell shows the best cycling performance. In Core/shell structures, the nanosheet shell covers the core structure form direct contact with the electrolyte and results in superior cycling stability while in case of pristine NF/NCS nanotube the core directly interact with the electrolytes and resulted in swelling and fracture. Moreover, our GNF/NCS/CNS core/shell structure gets two more advantages from graphene; the first is the excellent conductivity and mechanical flexibility properties of graphene backbone results in better response to expansions and contractions during charge discharge; and the second is the graphene layer covers the surface of nickel foam and avoids the oxidation of nickel foam collector by electrolytes and results in improved chemical stability of the composite^36^.

The comparative EIS studies were also conducted to understand the electrochemical reaction kinetics of the as-synthesized samples. Figure 5c and d describe the impedance responses of GNF/NCS/CNS core/shell, NF/NCS/CNS core/shell and NF/NCS electrodes measured at the open-circuit AC perturbation voltage of 5 mV potential in the frequency ranging from 100 KHz to 0.01 Hz. In the EIS plots, the Z’ intercept is related to the equivalent series resistance (R_ESR_), the high frequency semicircle diameter corresponds to the charge transfer resistance (R_ct_) and the more slope line in low frequency region is related to good capacitance behavior and low diffusion resistance^4, 37^. The magnified scale high frequency region EIS plot is shown in Fig. 5d; it clearly shows high semicircle for NF/NCS core electrodes and smallest semicircle for GNF/NCS/CNS core/shell electrode. The approximate R_ct_ values are 0.1, 0.155 and 0.32 Ω for GNF/NCS/CNS core/shell, NF/NCS/CNS core/shell and NF/NCS electrodes, respectively. Similarly, the approximate R_ESR_ values are 0.49, 0.5, and 0.52 Ω for GNF/NCS/CNS core/shell, NF/NCS/CNS core/shell and NF/NCS core electrodes, respectively. Moreover, the more slope line (Fig. 5c) of GNF/NCS/CNS core/shell structure demonstrating low diffusion resistance and ideal capacitance behavior of GNF/NCS/CNS core/shell. The lower impedance values of GNF/NCS/CNS core/shell could be attributed to the presence of porous NCS/CNS core/shell nanosheets which allow easy diffusion of ions and excellent conductive of graphene which served as super high way channel for electron transport in the composite.

**Figure 6:**
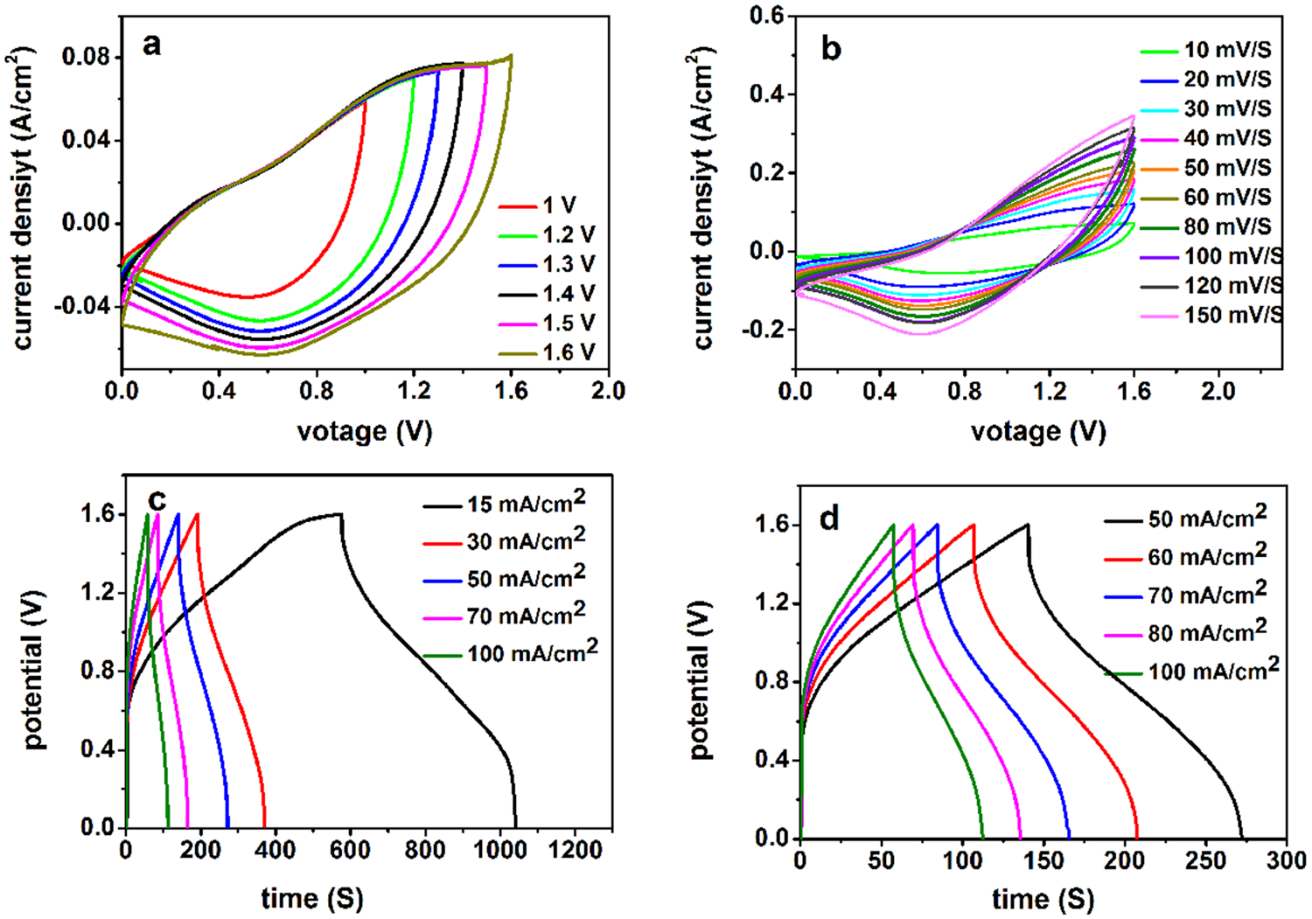
CV and GCD plots of GNF/NCS/CNS core/shell positive electrode and RGO negative electrode asymmetric device (**a**) CV plots at different potential windows of GNF/NCS/CNS core/shell at scan rate of 3 mV/S, (**b**) CV plots at different scan rates, (**c**) GCD plots at different current densities ranging from 15 to 100 mA/cm^2^ and (**d**) GCD plots at higher current densities ranging from 50 to 100 mA/cm^2^.

To investigate the practical application of GNF/NCS/CNS core/shell structure we assembled a prototype asymmetric electrode consisting of GNF/NCS/CNS core/shell as positive electrode and reduced graphene oxide (RGO) as the negative electrode. Asymmetric electrodes have been found to be an effective approach to meet the ever increasing demand of high energy density SCs, through providing a larger voltage window by combination of a carbon-based material as negative electrode with pseudocapacitive based materials as positive electrode. Cui *et al*. has shown RGO as excellent negative electrode for supercapcitor application, his group proposed as strong cation adsorption at the oxidized surface of rGO sheets make RGO excellent candidate for negative electrodes^42^. In asymmetric electrode the mass balance between the positive and negative electrodes is obtained by using a relation m_−_/m_+_ = (C_+_ΔV_+_)/(C_−_ΔV_−_)^43^, where, C_+_ and ΔV_+_ are correspondingly the mass specific capacitance and working voltage window of GNF/NCS/CNS core/shell electrode tested in the three electrode and similarly C_−_ and ΔV_−_ are correspondingly the mass specific capacitance and working voltage window of RGO electrode tested in three electrode. Hence, 44 mg/cm^2^ of RGO is required to balance 8 mg/cm^2^ of GNF/NCS/CNS core/shell positive electrode. Figure 6 illustrates the electrochemical performance of as-synthesized asymmetric device. Figure 6a shows CV plots as potential window increase from 1 V to 1.6 V at constant scan rate of 10 mV/s. When the operating voltage window is located at 1.0 V, a triangular like CV curve was obtained which implies an incomplete pseudocapacitive response while increasing the voltage window to 1.6 V oxidation and reduction peaks are observed in the CV curves indicating deeper redox reactions on the surface of electrode^43^; thus, clearly shows as our asymmetric device can support a wide potential window of 1.6 V. Figure 6b illustrates the CV plots of as synthesized asymmetric device at various scan rates ranging from 10 to 150 mV/s within potential window of 0 to 1.6 V. The CV geometry indicated obvious redox characteristics, which is quit consistent with pseudo capacitive property. Figure 6c and d illustrates the charge-discharge curves of as synthesized samples at different current densities within a potential window of 0 to 1.6 V. The GCD plots show symmetrical curves illustrating its fast reversibility; and the nonlinear shape of the GCD shows its pseudo capacitive property which is in a good agreement with the CV plots.

**Figure 7:**
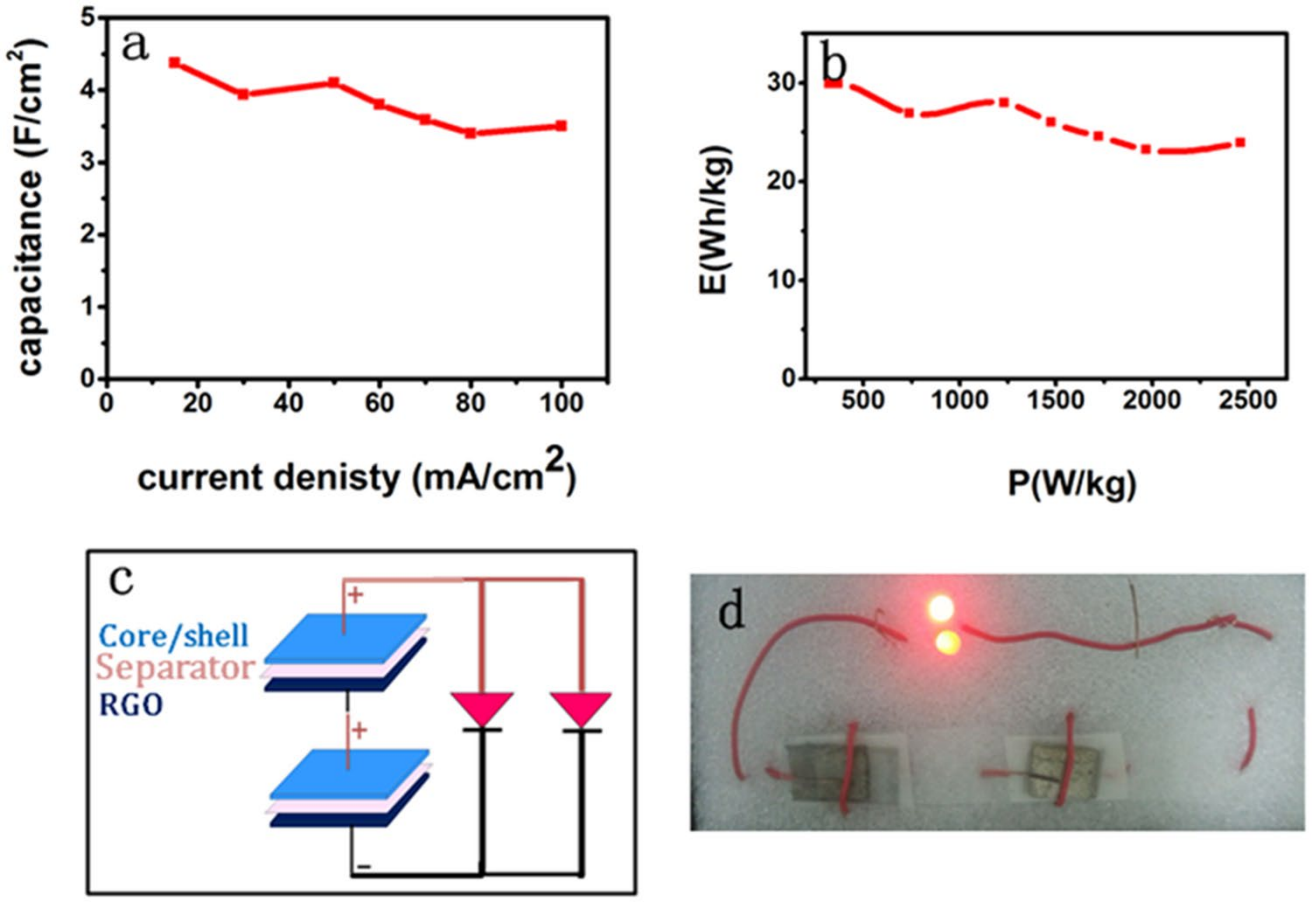
(**a**) Plot of areal capacitance as a function of current densities, (**b**) the Ragone plot of as synthesized electrode showing energy and power density relations, (**c**) Schematic illustration of two asymmetric supercapacitor in series with two LEDs in paralleled and (**d**) Two asymmetric supercapacitors connected in series and used to power on LEDs.

The capacitance of as fabricated electrode is calculated using equation (1); and the maximum areal capacitance obtain was 4.373 F/cm^2^ at current density of 15 mA/cm^2^. Figure 7a shows plot of areal capacitance as a function of current densities, interestingly, the capacitance decrease very little as current density increased from 15 to 100 mA/cm^2^ and illustrating excellent rate capability of 80%. The energy and power densities are calculated using a relation given in equation (4) and (5), respectively. Interestingly, the assembled asymmetric device shows excellent energy densities of 29.9 Wh/kg at 15 mA. Figure 7b shows the Ragone plot of as synthesized electrode showing good energy and power density relations, even at high power density of 2460.6 W/kg the energy density is still very high which is 23.93 Wh/kg. To insure practical application of as-synthesized electrode we conducted cycling performance test and interestingly the as synthesized electrode shows excellent cycling stability of 92% after 6000 cycles of charge discharge. Compared to recent reports listed in Table 2 our core/shell composite showed superior electrochemical performance. The main reason for excellent performance is related to the excellent and unique properties of graphene associated with the pours 3D core/shell structure which leads to increased active area and efficient contact between electrolyte ions and active materials. To demonstrate the potential of our asymmetric composite for real applications, we connected two asymmetric electrodes in series and used to power ON LEDs as shown in Fig. 7d. The two series devices successfully drive LEDs (0.8 V, 10 mA) for 35 minutes after full charged to 3.2 V.

In summary, Porous NCS/CNS core/shell structure was *in situ* grown on graphene decorated nickel foam current collector by using stepwise hydrothermal process. The excellent capacitive performance of our GNF/NCS/CNS core/shell structure is due to the contribution of high surface area graphene which supported 3D porous NCS/CNS core/shell structure. Moreover, the 3D porous shell structure leads to increase of active area and resulted in high areal capacity. Additionally, the graphene layer sandwiched between the NCS/CNS core/shell and nickel foam current collector serves as a super high way for electron transport and leads to better rate performance. Using these synergistic advantages of graphene and NCS/CNS core/shell 3D porous structure we got superior areal capacitance of 15.6 F/cm^2^ at current density of 10 mA/cm^2^, excellent cycling stability of 93% after 5000 of cycles and excellent rate capability of 74.36% as current increase from 10 to 100 mA/cm^2^. This kind of hybrid structure may hold great potential for high performance energy storage devices in the future.

## Methods

### Synthesis of Graphene/NCS/CNS core/shell structure

All the reagents used were analytical grade without farther purification. Prior to the synthesis process, nickel foam of area 2 × 4 cm^2^ was cleaned with deionized (DI) water and absolute ethanol (Et) and dried.

### Growth of 3D graphene on nickel foam

The pre cleaned nickel foam was placed in the quarz tube of CVD furnace and heated to 1023 °C in 80 minutes, maintained at 1023 °C for 20 minutes under argon (100 Sccm) gas atmosphere. Then, H_2_ (100 Sccm) and CH_4_ (2 sccm) gas were flew into the quartz tube for 90 minutes to grow 3D graphene layer on nickel foam (GNF). Finally, the sample was cooled down rapidly to room temperature with the gas protection of H_2_^35^.

### Growth of NCS nanotube on GNF

In this step, 1.855 g of CoCl_2_, 0.95 g of NiCl_2_, 0.72 g of urea and 60 ml of DI water were mixed by magnetic stirring until a transparent homogenous pink solution was formed and the solution was transferred to 80 ml Teflon-lined stainless steel autoclave. Then, the pre-prepared GNF was diagonal placed into the solution and the autoclave was sealed and kept in an oven at a temperature of 120 °C for 6 hour. After cooling down to room temperature, NCS nanoneedls precursor grown on GNF was washed with DI water and Et several times and dried at a temperature of 50 °C for 4 hours. Then, the resulted, GNF/NCS-precursor was hydrothermally treated in 80 ml Teflon-lined stainless steel autoclave containing a solution of 1.8 g of Na_2_S and 60 ml of DI water at temperatures of 180 °C for 8 hours. Then, after cooling down to room temperature and washing we get GNF/NCS nanotube.

### Growth of GNF/NCS/CNS core/shell structure

In this step, 0.5 g of CoCl_2_, 0.25 g of NiCl_2_, 1.2 g of urea and 12 ml of DI water, 12 ml Et and 12 ml of ethyl glycol were mixed by magnetic stirring until a transparent homogenous pink solution was formed and the solution was transferred to 60 ml Teflon-lined stainless steel autoclave and finally GNF/NCS was placed in the solution and it was hydrothermally treated at a temperature of 250 °C for 1 hours and after normally cooling down and washing we dried at a temperature of 50 °C for 4 hours and we got GNF/NCS/CNS core/shell.

### Preparation of RGO for negative electrode of asymmetric electrodes

It was prepared by hydrothermal reduction of GO; in detail, 120 mg of GO was immersed in 48 ml of DI water, then ultrasonicated for 5 hour at a temperature of 60 °C. Then, the solution was transferred to a 80 ml Teflon-lined stainless steel autoclave and heated at a temperature of 180 °C for 6 hrs, after cooling down to normal temperature the resulting sample is washed by vacuum filtration using DI water several times and dried at a temperature of 60 °C for 6 hr.

### Structural characterization

To study the morphology of as synthesized sample scanning electron microscopy (SEM) Hitachi S-4800 was used. To study how atoms are arranged in crystal structures, we conducted X-ray powder diffraction (XRD) patterns on a Rigaku Max-2200 X-ray diffractometer with monochromatized Cu_Kα_ radiation (λ = 0.1542 nm). X-ray photoelectron spectroscopy (XPS) was used to study chemical state of the elements in the composite. Raman spectroscopy was carried out by HR800 with an excitation wavelength of 633 nm to study graphene on the nickel foam.

### Electrochemical measurements

The electrochemical properties of the as-synthesized sample were tested by conducting cyclic Voltammetry (CV), galvanostatic charge-discharge (GCD) and electrochemical impedance spectroscopy (EIS). All electrochemical performance measurements were carried out using electrochemical workstation (CS 2350 Corrtest, Wuhan, China) using 3 M KOH aqueous electrolytes. In a three-electrode system measurement, the as-synthesized active material was used as a working electrode; Platinum plate and Ag/AgCl with saturated KCl are used as a counter and reference electrodes, respectively. A 1 × 1 cm^2^ area GNF/NCS/CNS core/shell sample was sandwiched between two pieces of nickel foams and pressed at pressure of 3 MPa and used as working electrode. Moreover, we also tested the electrochemical performance of assembled prototype working device. In this case, we used GNF/NCS/CNS core/shell as positive electrode and RGO as negative electrode. The RGO negative electrode was prepared by coating a mixture of RGO, acetylene black and poly (tetrafluoroethylene) in a weight ratio of 80: 10: 10 on nickel foam. Then, the electrode was dried for 12 hour at 60 °C in a vacuum. The average areal specific capacitance and mass specific capacitance values in three electrode measurement were calculated from GCD curves, using equations (1) and (2)^44, 45^:

1 $${C}_{A}=\frac{I\times {\rm{\Delta }}t}{S\times {\rm{\Delta }}V}$$

2 $${C}_{s}=\frac{I\times {\rm{\Delta }}t}{m\times {\rm{\Delta }}V}$$

Where *C*_*A*_(F/cm^2^), *C*_*S*_(F/g), I(A), t(S), m(g), S(cm^2^) and Δ*V*(V) are specific capacitance, areal capacitance, discharge current, discharge time, mass of active material, area of active electrode and potential window for charge discharge, respectively. On the other hand, the specific capacitance of two-electrode system is calculated using the relation given by refs 46 and 47:

3 $${C}_{cell}=\frac{{\rm{I}}\times {\rm{\Delta }}{\rm{t}}\,}{{\rm{\Delta }}{\rm{V}}\times {\rm{m}}^{\prime} }$$

Where C_cell_ is the specific capacitance of an asymmetric cell, I is discharge current of the cell, ΔV is the operating voltage window of the cell, m’ is sum mass of active materials on both electrode (m’ = m_−_ + m_+_, where m_-_ is total mass of RGO and m_+_ is total mass of GNF/NCS/CNS active material used) after obtaining the balanced mass of the two electrodes. Energy density and power density are key parameters to measure the practical performance of electrodes. So, the energy and power densities are calculated using the following relations:

4 $$E=\frac{\,{C}_{cell}{{\rm{V}}}^{2}}{2\times 3600}$$

5 $$P=\frac{E\times 3600}{t}$$

Where, E, P, V and t are energy density, power density, charging potential widow and discharge time, respectively^44^.
